# Alveolar Macrophages Participate in the Promotion of Influenza Virus Infection by Aflatoxin B1 at an Early Stage

**DOI:** 10.3390/toxins15010067

**Published:** 2023-01-12

**Authors:** Yuhang Sun, Zhaoran Yao, Miao Long, Ying Zhang, Kehe Huang, Lin Li

**Affiliations:** 1 College of Animal Science & Veterinary Medicine, Shenyang Agricultural University, Shenyang 110866, China; 2 Key Laboratory of Livestock Infectious Diseases, Ministry of Education, Shenyang Agricultural University, Shenyang 110866, China; 3 Department of Animal Nutrition and Immunology, College of Veterinary Medicine, Nanjing Agricultural University, Nanjing 210095, China

**Keywords:** aflatoxin B1, swine influenza virus, alveolar macrophage, macrophage polarization, macrophage depletion

## Abstract

Aflatoxin B1 (AFB1), one of the most common environmental mycotoxin contaminations in food and feed, poses significant threats to human and animal health. Our previous study indicated that even non-toxic AFB1 concentrations could promote influenza virus replication and induce influenza virus-infected alveolar macrophages polarizing from M1 (immunostimulatory phenotype) to M2 (immunosuppressive phenotype) over time. However, whether AFB1 promotes influenza replication via modulating the polarization of alveolar macrophages is unknown. Here, we specifically depleted alveolar macrophages using clodronate-containing liposomes in swine influenza virus (SIV)-infected mice to explore the mechanism the promotion of SIV replication by AFB1. The results show that the depletion of alveolar macrophages significantly alleviated the AFB1-induced weight loss, inflammatory responses, and lung and immune organ damage of the SIV-infected mice after 14 days and greatly diminished the AFB1-promoted SIV replication. In contrast, the depletion of alveolar macrophages did not alleviate the AFB1-induced weight loss, and lung and immune organ damage of the SIV-infected mice after 28 days and slightly diminished the AFB1-promoted SIV replication. Collectively, the data indicate that alveolar macrophages play a crucial role the promotion of SIV infection by AFB1 in the early rather than late stage, and AFB1 can promote SIV replication by inducing alveolar macrophages to polarize towards M1 macrophages. This research provides novel targets for reducing the risk of AFB1-promoted influenza virus infection.

## 1. Introduction

Mycotoxin contaminations are common and prevalent all over the world. Aflatoxin B1 (AFB1), derived from *Aspergillus flavus*, is one of the most common and routinely detected mycotoxin contaminants in moldy food, livestock feed, and their raw materials (such as corn, peanut meal, etc.) [1]. Currently, 37 countries worldwide have set limits for AFB1 in food and cereals. According to the latest feed hygiene standards issued by the U.S. Food and Drug Administration (FDA), European Union, and the Chinese Ministry of Agriculture, the limit of AFB1 in commercial pig feed is 50 μg/kg. However, a survey in 2021 from China showed that the positive rate of AFB1 in new corn was 7–91% from eight major producing areas, and the positive rate of AFB1 in peanut meal was even 100%; the positive rate of AFB1 in commercial pig feed was 21%. Even non-toxic AFB1 (20 to 40 μg/kg) exposure was confirmed to significantly promote viral infection in pigs [2,3]. Therefore, it is of great importance to understand and determine the toxicity and mechanism of AFB1 in pigs.

AFB1 not only has hepatotoxicity and nephrotoxicity but also has severe immunotoxicity [4]. Similar to ochratoxin (OTA; long-term rather than short-term OTA exposure induces immunosuppression [5,6]) and deoxynivalenol (DON; a low-dose of DON induces inflammation but a high-dose of DON promotes immunosuppression [7]), AFB1 exhibits two-way immunotoxicity, including immunostimulation and immunosuppression. However, inflammation (also called immunostimulation) or immunosuppression depends on various factors, including AFB1 exposure dose, exposure time, species, sex, age, and the presence or absence of immunologic stimulants, and so on [8]. In general, short-term or low-dose AFB1 exposure can induce inflammation, but long-term or high-dose AFB1 exposure promotes immunosuppression. As previously reported, 0.01 to 0.1 μg/mL AFB1 exposure markedly increased the levels of TLR4 in lymphocytes and monocytes [9], while 4 to 8 μg/mL AFB1 exposure significantly suppressed the proliferation of splenocytes and decreased the IL-2 content in cells [10]. Moreover, previous studies indicated that AFB1 exposure impaired the proliferation of pig alveolar macrophages (PAMs; 3D4/21) [11], and AFB1 (>16 μmol/mL) exposure for 3 h decreased the bacterial uptake by PAMs [12]. Among mammalian species, swine is most sensitive to AFB1, and AFB1 exposure can lead to inflammation or immunosuppression in pigs at different stages, even increasing their susceptibility to some infectious diseases, bringing great harm to pig production.

The swine influenza virus (SIV), a single-stranded, segmented RNA virus with a capsule, belongs to *Orthomyxoviridae*, a genus of *influenza viruses*. It is one of the most widespread influenza viruses in the world, causing severe zoonosis and even death [13]. Pigs are excellent animal models for the study of influenza. On the one hand, pigs are highly similar to humans in tracheobronchial structure, lung physiology and size, and airway submucosal glands, and the clinical symptoms and mechanisms of the disease are similar to humans. On the other hand, the same influenza subtypes (H1N1 and H3N2) are prevalent in pigs and humans. In addition to pigs and humans, the SIV can also infect birds and ferrets, among which pigs are the most important vector of transmission, known as the “mixer” of the influenza virus, which plays an important role in influenza epidemics and related studies. Different to the highly pathogenic avian influenza virus, the SIV is a mild virus, and its infection degree is affected by a variety of environmental and nutritional factors, including mycotoxin exposure. Therefore, SIV infection can be studied from the perspective of regulating environmental and nutritional factors. In general, low-level pathogenic swine influenza can occur throughout the year, with a low mortality rate (<1%) but a high infection rate (up to 100%). Infected pigs generally recover in about 7 days, and the epidemic ends in 2 to 3 weeks. However, our previous study confirmed that non-toxic AFB1 exposure could promote SIV replication (relative SIV M protein mRNA levels increased four to five times) in mice and several in vitro cultured cell lines, including PAMs, and low-dose and long-term AFB1 exposure also modulated the polarization of alveolar macrophages, switching from M1 to M2 macrophages [2,3,14], inducing immunosuppression; however, the mechanism of action remains unclear.

Alveolar macrophages are the body’s first line to defend against influenza virus infection [15]. According to the inflammatory state, alveolar macrophages can polarize into M1 or M2 macrophages. M1-type alveolar macrophages mainly secrete pro-inflammatory cytokines, which can resist pathogen invasion and promote tissue damage. M2-type alveolar macrophages mainly secrete anti-inflammatory cytokines, which can reduce inflammatory responses and repair damaged tissues. The phenotype in which macrophages exhibit two different functions of M1 and M2 is called macrophage polarization [16]. Macrophage polarization is an essential mechanism for maintaining immune homeostasis and participates in immune regulation. Previous studies have shown that macrophages exhibit pro-inflammatory M1 and anti-inflammatory M2 phenotypes with different functions under different stimuli [17,18]. A variety of polarization markers, cytokines, and growth factors, including TNF-α, IL-10, Arg-1, TGF-β, CD86, CD206, and MRC-1, jointly determine the final polarization state of macrophages [19]. Our previous results demonstrated that long-term exposure to AFB1 can induce the phenotypic polarization of SIV-infected alveolar macrophages from M1 to M2, resulting in immunosuppression [2]. However, whether alveolar macrophages play a vital role in the AFB1-promoted influenza infection and when the alveolar macrophages play a crucial role are unknown.

Therefore, based on the relevant literature and previous research results, we present a hypothesis that low-dose AFB1 exposure could promote SIV replication by modulating the polarization of alveolar macrophages to M1 or M2 macrophages. In this present study, we conduct two experiments, using clodronate-containing liposomes to establish a mouse model of alveolar macrophage depletion for 14 and 28 days, respectively, in order to investigate the mechanism of AFB1-promoted SIV infection. The results provide new insights and targets for the prevention and treatment of influenza, and also provide new ideas for further research on the causes of the increase in subclinical infection.

## 2. Results

### 2.1. Alveolar Macrophage Depletion Diminished the AFB1-Induced Weight Loss and Lung Damage of the SIV-Infected Mice after 14 Days

**Figure 1:**
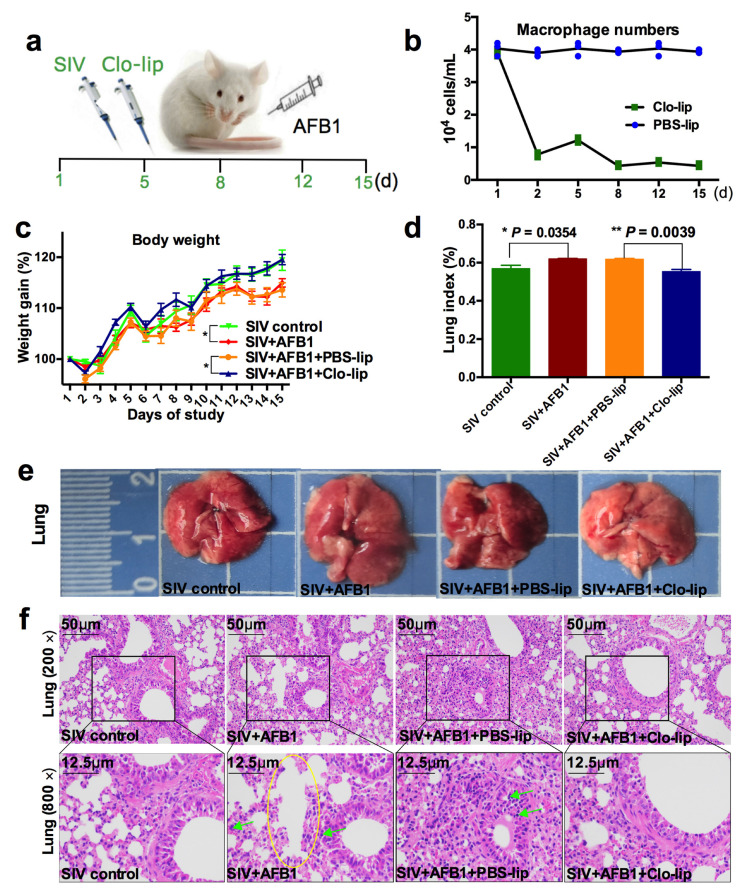
Weight gain and lung damage of the SIV-infected mice exposed to AFB1 after alveolar macrophage depletion for 14 days. (**a**) The experimental flow chart. SIV, PBS liposomes, and clodronate-containing liposomes were given on days 1, 5, 8, 12, and 15, and AFB1 was injected every day. (**b**) The result of the pre-trial experiment of alveolar macrophage depletion. (**c**) Weight gain. Daily gain is expressed as a percentage of body weight to original body weight, * *p* < 0.05. (**d**) Lung index is calculated by a percentage of lung mass to body weight, * *p* < 0.05, ** *p* < 0.01. (**e**) Lung pictures. The scale unit is centimeters. (**f**) Lung HE staining pictures. The yellow circle indicates large fused alveoli, and the green arrows indicate infiltrating inflammatory cells.

As reported previously, AFB1 can promote SIV replication and modulate the switching of alveolar macrophages from M1 towards M2 [2]. To investigate whether alveolar macrophages participate in the promotion of SIV replication by AFB1, we tried to deplete the alveolar macrophages (Figure 1a). The results demonstrate that macrophages almost faded away after three Clo-lip injections (Figure 1b), suggesting that the alveolar macrophages were depleted. Next, the weight (Figure 1c) and lung indices (Figure 1d) were measured to assess the role that alveolar macrophages played. The results show that the weight gain decreased, but the lung index increased in Group B relative to Group A; meanwhile, compared to Group C, the weight gain increased (Figure 1c), but the lung index decreased (Figure 1d) in Group D. Subsequently, lung tissues were collected, captured by a camera, and stained by HE. As shown in Figure 1e,f, after AFB1 injection (Group B), the lung of mice (Group A) was enlarged, several tiny alveoli were ruptured and fused to form large alveoli to achieve compensatory function, and inflammatory cells were significantly increased. In contrast, compared to PBS-lip injection (Group C), the mice had less enlarged lungs and fewer inflammatory cells but no noticeable improvement in the alveolar structure after CLP injection (Group D).

### 2.2. Alveolar Macrophage Depletion Diminished the AFB1-Induced Inflammation and Immune Organ Damage of the SIV-Infected Mice after 14 Days

**Figure 2:**
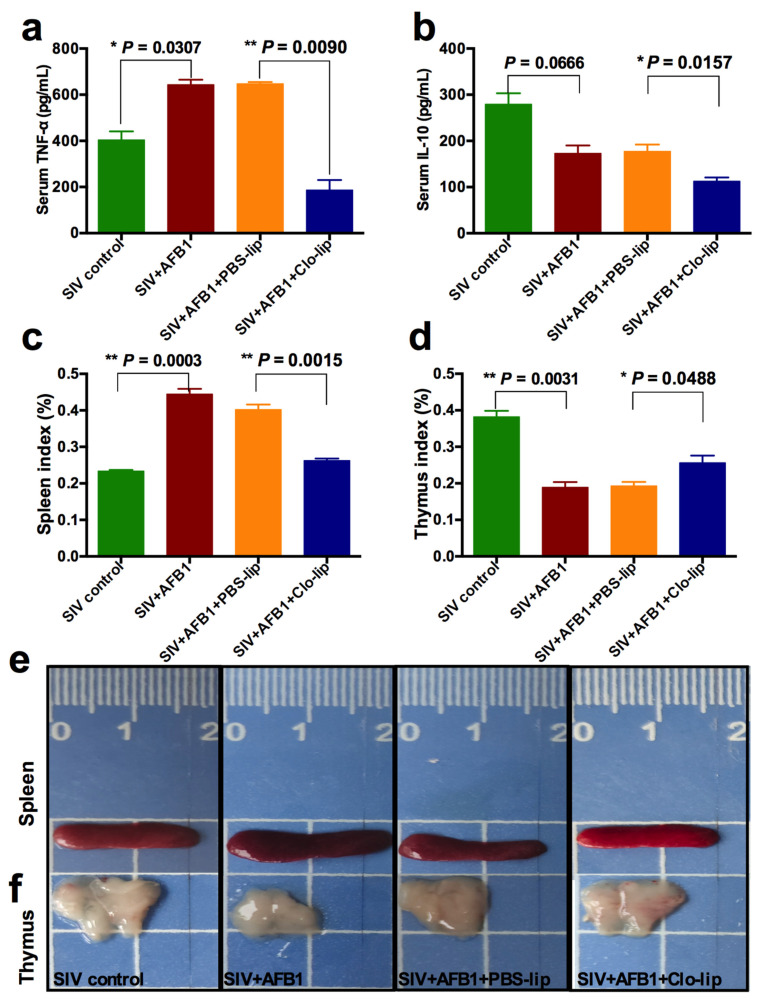
Serum cytokine levels and immune organ damage of the SIV-infected mice exposed to AFB1 after alveolar macrophage depletion for 14 days. (**a**) TNF-α content; (**b**) IL-10 content; (**c**) spleen index; and (**d**) thymus index; * *p* < 0.05, ** *p* < 0.01. (**e**) Spleen and (**f**) thymus pictures. The scale unit is centimeters.

**Figure 3:**
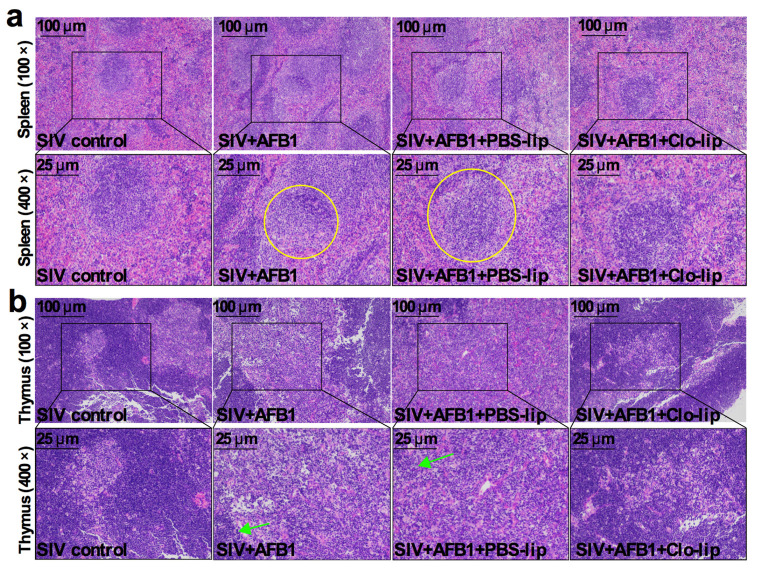
Immune organ damage of the SIV-infected mice exposed to AFB1 after alveolar macrophage depletion for 14 days. (**a**) Spleen and (**b**) thymus HE staining pictures. Yellow circles show the disorganized white pulp of the spleen, and green arrows represent the disorganized thymus cortex.

TNF-α and IL-10, the M1 and M2 macrophage polarization markers, were detected to study the inflammatory states in mice. As shown in Figure 2a,b, the TNF-α level (Figure 2a) was markedly enhanced in Group B relative to Group A, but no noticeable change was observed in IL-10 (Figure 2b); at the same time, TNF-α and IL-10 levels markedly decreased in Group D relative to Group C (Figure 2a,b). The data indicate that AFB1 exposure increases the inflammatory responses in the SIV-infected mice and promotes the polarization of alveolar macrophages to M1, and that the depletion of alveolar macrophages significantly diminishes the inflammatory responses after 14 days. Next, immune organ damage was also assessed. The results show that the spleen index significantly increased (Figure 2c), but the thymus index markedly reduced in Group B relative to Group A (Figure 2d). Consistently, the spleen was swollen (Figure 2e), and the thymus was atrophic (Figure 2f) after the AFB1 injection. Meanwhile, after the AFB1 injection, the white pulp of the spleen (Figure 3a) and thymus cortex (Figure 3b) were disorganized. As expected, alveolar macrophage depletion using Clo-lip reversed all the above changes (Figure 2c–f and Figure 3a,b), suggesting that alveolar macrophage depletion could play a key role in AFB1-promoted SIV infection after 14 days.

### 2.3. Alveolar Macrophage Depletion Greatly Diminished the AFB1-Increased SIV Replication in the SIV-Infected Mice after 14 Days

**Figure 4:**
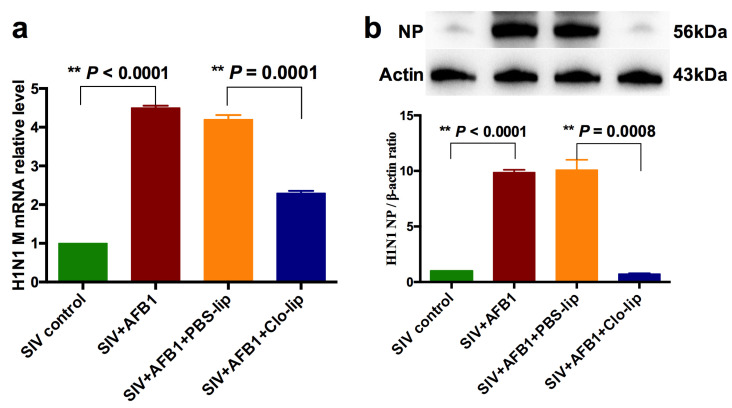
SIV replication of the SIV-infected mice exposed to AFB1 after alveolar macrophage depletion for 14 days. (**a**) SIV M mRNA; (**b**) SIV NP level; ** *p* < 0.01.

To further study the role that alveolar macrophages played, SIV replication was detected. The results demonstrate that AFB1 extremely markedly enhanced SIV M mRNA and NP protein levels but markedly decreased in SIV + AFB1 + Clo-lip relative to SIV + AFB1 + PBS-lip groups (Figure 4a,b), suggesting that the depletion of alveolar macrophages could reverse the AFB1-promoted SIV replication.

### 2.4. Alveolar Macrophage Depletion Did Not Alleviate the AFB1-Induced Weight Loss and Lung Damage of the SIV-Infected Mice after 28 Days

**Figure 5:**
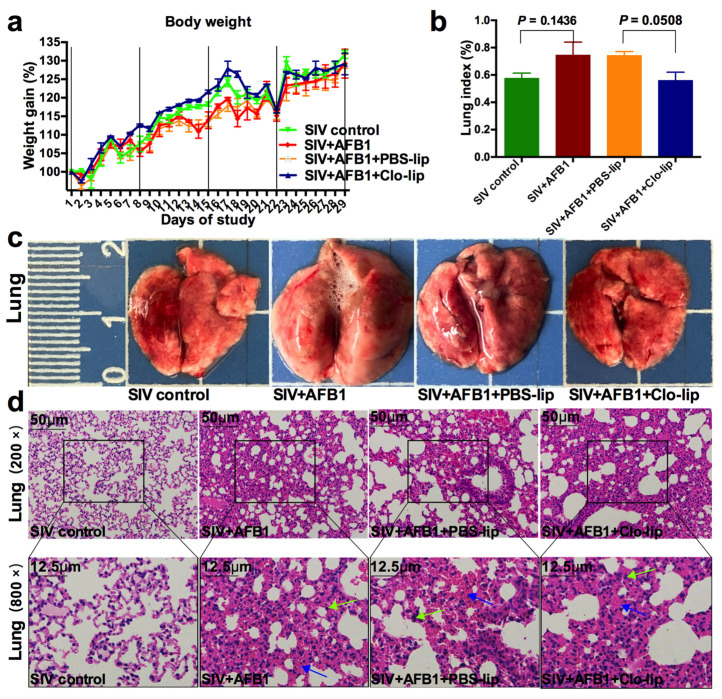
Weight gain and lung damage of the SIV-infected mice exposed to AFB1 after alveolar macrophage depletion for 28 days. (**a**) Weight gain; (**b**) Lung index; (**c**) Lung pictures. The scale unit is centimeters. (**d**) Lung HE staining pictures. Green arrows indicate infiltrating inflammatory cells, and blue arrows indicate hyperemia of the alveolar walls.

To understand whether AFB1 promotes SIV replication by modulating the switching of alveolar macrophages from M1 towards M2, the macrophage depletion experiment was extended to last for 28 days. The results demonstrate that there were no significant differences in the weight gain and the lung index between any two groups among the four groups (Figure 5a,b). As shown in Figure 5c,d, the lung damage was severe; there were a lot of infiltrating inflammatory cells. The alveolar walls were severely hyperemic, but no significant changes were observed between SIV + AFB1 + Clo-lip and SIV + AFB1 + PBS-lip groups, suggesting that alveolar macrophages can fail to play a vital role in the late stage of SIV infection.

### 2.5. Alveolar Macrophage Depletion Diminished the AFB1-Induced Inflammation but Did Not Alleviate the Immune Organ Damage of the SIV-Infected Mice after 28 Days

**Figure 6:**
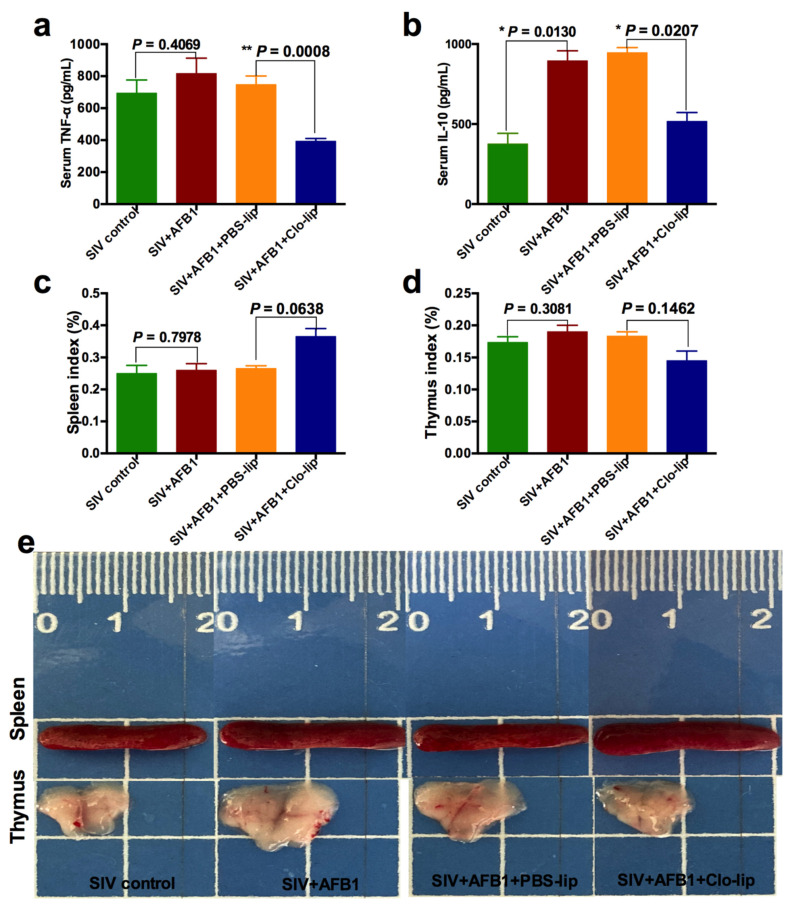
Serum cytokine levels and immune organ damage of the SIV-infected mice exposed to AFB1 after alveolar macrophage depletion for 28 days. (**a**) TNF-α content; (**b**) IL-10 content; (**c**) spleen index; and (**d**) thymus index; * *p* < 0.05, ** *p* < 0.01. (**e**) Spleen and thymus pictures. The scale unit is centimeters.

**Figure 7:**
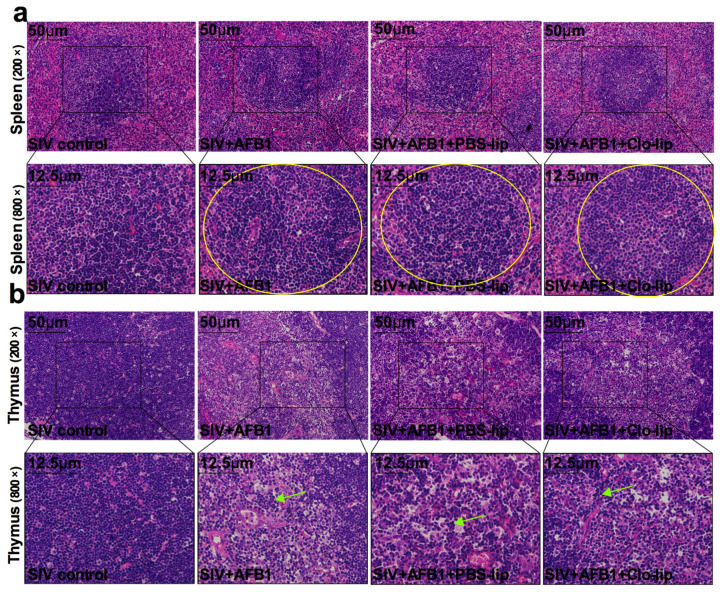
Immune organ damage of the SIV-infected mice exposed to AFB1 after alveolar macrophage depletion for 28 days. (**a**) Spleen and (**b**) thymus HE staining pictures. Yellow circles show the disorganized white pulp of the spleen, and green arrows represent the disorganized thymus cortex.

Similar to 14 days, TNF-α and IL-10 in serum were also detected after 28 days to investigate the inflammatory state and the macrophage polarization state in mice. The results demonstrate that no noticeable change was observed in the TNF-α level. Still, the IL-10 level was significantly enhanced in Group B relative to Group A (Figure 6a,b), suggesting that AFB1 promoted the polarization of SIV-infected macrophages to the M2 phenotype after 28 days. On the contrary, TNF-α and IL-10 levels together decreased in Group D relative to Group C (Figure 6a,b). The data suggest that alveolar macrophage depletion diminished the inflammatory responses and prevented macrophages from polarizing to the M2 phenotype. Next, immune organ damage was also assessed. Unexpectedly, there were no significant differences in the spleen index and the thymus index (Figure 6c,d) between Groups C and D. Consistently, there were no apparent changes in the appearance of the spleen and thymus (Figure 6e), as well as the HE staining pictures (Figure 7a,b), between Groups C and D. Taken together, our data suggest that the depletion of alveolar macrophages could not work in AFB1-promoted SIV infection in the late stage, and the polarization of macrophages to M1 but not M2 might be the critical mechanism of the AFB1-promoted SIV infection.

### 2.6. Alveolar Macrophage Depletion Slightly Diminished the AFB1-Increased SIV Replication in the SIV-Infected Mice after 28 Days

**Figure 8:**
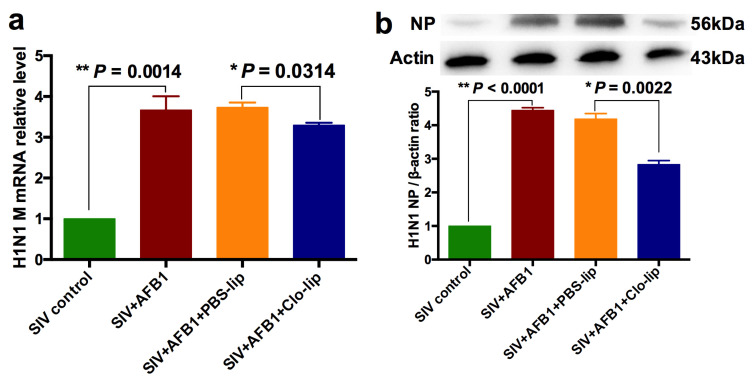
SIV replication of the SIV-infected mice exposed to AFB1 after alveolar macrophage depletion for 28 days. (**a**) SIV M mRNA; (**b**) SIV NP; * *p* < 0.05, ** *p* < 0.01.

SIV replication was also detected after 28 days. The results demonstrate that AFB1 extremely markedly enhanced SIV M mRNA and NP protein levels (Figure 8a,b). At the same time, they slightly decreased in SIV + AFB1 + Clo-lip relative to SIV + AFB1 + PBS-lip groups (Figure 8a,b), suggesting that alveolar macrophage depletion could slightly alleviate the AFB1-promoted SIV replication at a late stage.

## 3. Discussion

Our present data indicate that alveolar macrophages could play a key role the promotion of SIV infection by AFB1 in the early rather than late stage, which is consistent with previous studies demonstrating that alveolar macrophages participate in influenza infection at an early stage [15,20].

Our previous experiment also demonstrated that AFB1 promoted the switching of SIV-infected alveolar macrophages towards M1 at 14 days and towards M2 at 28 days [2], while this present study demonstrates that alveolar macrophages play a vital role in the promotion of SIV infection by AFB1 at an early rather than late stage, suggesting that the polarization of macrophages to M1 but not M2 might be the crucial mechanism of AFB1-enabled SIV infection. The SIV is always considered an immunosuppressive virus because SIV infection can induce immunosuppression, but the inflammatory state in SIV-challenged animals depends on the time of SIV infection. A previous mouse study indicated that mice infected with the influenza virus for two days exhibited the excessive infiltration of macrophages and increased Th1 cytokines, demonstrating that the mice had a primarily Th1 response [21]; an in vitro mouse study indicated that the influenza virus could induce the dynamic polarization of mouse alveolar macrophages from M1 to M2 over time [17]. Meanwhile, AFB1 also has two-way immunotoxicity, demonstrating that short-term exposure to AFB1 induces an inflammatory response, but long-term exposure to AFB1 promotes immunosuppression [8]. Therefore, we concluded that short-term exposure to AFB1 could promote SIV infection by increasing M1 macrophages, which is consistent with our results demonstrating that the depletion of alveolar macrophages significantly alleviated the AFB1-promoted SIV infection after 14 days.

Why did alveolar macrophage depletion not significantly alleviate the AFB1-promoted SIV infection after 28 days? Our previous study showed that AFB1 promoted the switching of SIV-infected alveolar macrophages from M1 to M2 after 28 days [2]. As described above, M2 alveolar macrophages mainly secrete anti-inflammatory cytokines, which can reduce the inflammatory response and promote tissue repair. A previous study indicated that AFB1 could directly suppress macrophages, especially M2 macrophages, to disrupt the immune function [22]. Consistently, another previous study indicated that M2 alveolar macrophages produced anti-inflammatory cytokines, thereby inhibiting influenza-mediated lethal inflammation [23], suggesting that the inflammatory response in alveolar macrophages faded to repair lung tissues. However, SIV replication persists and increases up to day 28, meaning that M2 alveolar macrophages failed to defend against the influenza virus infection. Therefore, the depletion of alveolar macrophages did not significantly alleviate the AFB1-promoted SIV infection and tissue injuries after 28 days, and AFB1 might not promote SIV infection by promoting the polarization of M2 alveolar macrophages.

In fact, polarization from M1 to M2 macrophage is an excessively inflammatory process, suggesting that AFB1 aggravated the influenza-induced inflammation. Consistently, a lot of previous studies indicated that the influenza virus infection promotes inflammation and tissue injury, and the inhibition of inflammation can effectively avoid influenza-induced lethality [24,25,26,27]. In these studies, TLR4 was considered a critical target for inhibiting inflammation. Interestingly, TLR4 has been proven to modulate the polarization of M1 macrophages in previous reports [28,29,30]. Furthermore, other signals, including Akt [31], PI3K [32], and mTOR [33], were confirmed to participate in the modulation of macrophage polarization (M1/M2). For example, TLR4 activated the PI3K–Akt pathway to induce M2 macrophage polarization [34], and the PTEN/PI3K/Akt pathway activates M2 macrophage polarization [35]. Thus, in future, it is necessary to further explore which pathways play a critical role in the polarization of M1 macrophages induced by the promotion of influenza virus replication by AFB1. On the other hand, except for reducing M1 macrophages, raising “new-born” alveolar macrophages could be helpful to restore macrophage function. As expected, an experiment on the overexpression of the granulocyte-macrophage colony-stimulating factor (GM-CSF) proved that increased alveolar macrophages could prevent mortality induced by the influenza virus [36]. Therefore, in future, experiments on the overexpression of alveolar macrophages could be needed to study the prevention of and treatment for the AFB1-promoted influenza virus infection.

## 4. Conclusions

From what has been discussed above, alveolar macrophages participate in the promotion of the H1N1 virus infection by AFB1 at an early stage. Therefore, the early prevention and control of the AFB1-promoted influenza virus replication, eliminating inflammation in alveolar macrophages, or recruiting an adequate number of alveolar macrophages may be effective ways to control the outbreak of the swine influenza virus. Our study provides new insights and targets for the prevention and treatment of influenza.

## 5. Materials and Methods

### 5.1. Viruses and Reagents

The H1N1 virus (A/Swine/Guangxi/18/2011) was stored in our laboratory and propagated in MDCK (Madin-Darby canine kidney) cells. MDCK cells were cultured in DMEM at 37 °C with 5% CO_2_. The virus was cultured in serum-free DMEM, and its titers were measured by the (tissue culture infectious dose 50) TCID_50_ method.

AFB1 (Sigma-Aldrich, 1162-65-8, St. Louis, MO, USA) was dissolved with (dimethylsulfoxide) DMSO and further diluted with phosphate-buffered saline (PBS) for injection. Clodronate-containing liposomes (CLPs) and PBS liposomes were purchased from Shanghai Qifa Experimental Reagent Co., Ltd. (Shanghai, China). CLPs, a lung macrophage scavenger, can specifically deplete alveolar macrophages by nasal injection [37,38,39]. As the kit instructions described, after one CLP injection, alveolar macrophages decrease by about 80% and almost fade away after multiple CLP injections.

### 5.2. Animal Experiment Design

A total of forty BALB/c mice weighing 18 to 20 g were provided by Liaoning Changsheng Biological Technology Co. Ltd (Shenyang, China). After one-week adaptation, 40 mice were randomly assigned into 4 groups (10 mice/group): (A) SIV control; (B) SIV + AFB1; (C) SIV + AFB1 + PBS liposomes (PBS-lip); (D) SIV + AFB1 + CLP (Clo-lip). All mice from 4 groups were given 1000 TCID_50_ H1N1 virus (100 µL virus dilution, TCID_50_ = 10^4.5^) on days 1, 5, 8, 12, 15, 19, 22, 26, and 29 of this experiment via nasal injection. The mice from groups B, C, and D were injected (i.p.) with AFB1 (40 μg/kg b.w.) every day. The mice from groups C and D were given 50 µL PBS liposomes and CLPs, respectively, on days 1, 5, 8, 12, 15, 19, 22, 26, and 29 days of this experiment via nasal injection. On days 15 and 29 of this experiment, five mice from each group were sacrificed, respectively, and then the blood, lung, spleen, and thymus tissues were collected.

### 5.3. Quantitative Real-Time PCR (QRT-PCR)

As described by our previous study [5], total RNA was extracted from the lungs to determine the relative SIV M mRNA. QRT-PCR was carried out using the SYBR Green real-time PCR kit (TaKaRa, Dalian, Japan) and the ABI Prism Step One Plus detection system (Applied Biosystems, Waltham, MA, USA). Finally, the SIV M mRNA level was measured by calculating its relative level to a reference gene (GAPDH).

### 5.4. Western Blots

Total proteins were extracted from the lungs to detect the SIV NP expression level. In brief, the PVDF membrane containing the protein was continuously co-incubated with 5% milk or bovine serum albumin (BSA) for 2 h, a specific anti-NP antibody (Abcam, ab128193, London, UK) for 12 h at 4 °C, and an anti-mouse secondary antibody (Elabscience, Wuhan, China) for 1.5 h at room temperature (RT), respectively. Finally, a light chemiluminescence kit (EpiZyme, Shanghai, China) was used to recognize the bound antibodies. Image J software read the NP expression level relative to β-actin.

### 5.5. TNF-α and IL-10 Measurements

On days 15 and 29 of this experiment, five mice from each group were sacrificed, respectively, and then the serum was collected. Subsequently, the TNF-α and IL-10 contents in serum were detected by ELISA kits (Jiancheng, Nanjing, China).

### 5.6. Histopathological Analysis

Lungs, spleens, and thymuses were fixed in 4% formalin. Then, the samples were sectioned (4 µm thick) for further HE staining. An optical microscope was used to observe and snap the tissue damage and pathological changes; lung damage was assessed based on the amount of edema, alveolar rupture, and cellular infiltration; and spleen and thymus damage was evaluated according to the amount of white pulp atrophy, structure disorder, and bleeding.

### 5.7. Statistical Analysis

GraphPad Prism 7 was used to analyze all data, and the results were expressed using the mean ± SEM. For comparison between the two groups, a Student’s *t*-test was used. * *p* < 0.05 was statistically significant.

## Data Availability

The data presented in our study are available in this article.
